# Time-space characteristics of emergency medical service attendance and layperson naloxone administration during non-fatal opioid overdoses in Rhode Island: A retrospective, event-level analysis

**DOI:** 10.1016/j.annepidem.2025.02.009

**Published:** 2025-02-24

**Authors:** Joseph G. Rosen, Melissa Basta, Kristen St. John, Benjamin D. Hallowell, Maxwell S. Krieger, Lila Flavin, Ju Nyeong Park

**Affiliations:** a Department of Medicine, Warren Alpert Medical School, Brown University, Providence, RI, USA; b Division of General Internal Medicine, Rhode Island Hospital, Providence, RI, USA; c Substance Use Epidemiology Program, Rhode Island Department of Health, Providence, RI, USA; d Department of Epidemiology, School of Public Health, Brown University, Providence, RI, USA; e Department of Addiction Medicine, Warren Alpert Medical School, Brown University, Providence, RI, USA

**Keywords:** EMS, Opioids, People who use drugs, Overdose reversal, Non-fatal drug overdose, United States

## Abstract

**Purpose:**

As the opioid overdose crisis worsens in the United States (U.S.), emerging scholarship has uncovered time-and-place variations in substance use and overdose response efforts in community settings. Building on this work, we characterized spatio-temporal attributes of naloxone administration during non-fatal opioid overdoses attended by laypersons and emergency medical services (EMS) over a three-year period.

**Methods:**

Leveraging EMS encounter data across Rhode Island between January 2020 and December 2022, we quantified hour-by-hour variations in EMS deployment locations for non-fatal opioid-involved overdoses among adults (aged 18 + years). We used multivariable Poisson regression with robust standard errors to identify spatiotemporal patterns in EMS-attended overdoses by location type and evidence of layperson naloxone administration during these events.

**Results:**

Of the 5377 EMS non-fatal opioid overdose encounters, most occurred in residential housing (61.1 %) and outdoor public spaces (19.3 %). We identified substantial time-space variations in non-fatal overdoses, with EMS deployments to residential housing clustering in non-daylight hours (5:00PM-8:59AM) and to outdoor public spaces in daylight hours (9:00AM-8:59PM). Documented naloxone intervention by laypersons prior to EMS arrival was uncommon (10.6 %) but was most pronounced in overdoses occurring in residential housing and the early afternoon (1:00PM-4:59PM).

**Conclusions:**

Despite the clustering of non-fatal opioid overdoses in housing environments, we identified substantial within-location variations in overdose-related EMS encounters over time and place.

## Introduction

Opioid-involved overdoses in the United States (U.S.) have sky-rocketed in recent years, driven in large part by the introduction of fentanyl and its potent synthetic analogs into the illicit drug supply [6, 7]. Despite growing public awareness and possession of naloxone, an opioid antagonist that can safely and effectively reverse the effects of overdose in seconds [8,18,31], opioid overdose rates have risen exponentially [17], representing a growing fraction of ambulance and other emergency medical service (EMS) callouts [4,5,19]. Beyond the human cost of opioid overdose, which is responsible for over 80,000 deaths annually in the U.S. Mattson et al., [27,35], annualized financial costs of fatal and non-fatal opioid overdoses are estimated to exceed $1 trillion [12]. The magnitude of the U.S. opioid overdose crisis, thus, presents an unprecedented challenge to medical resource management in urban and rural settings.

Opioid overdose response efforts are further complicated by time- and-place variations in substance use and, subsequently, overdose risk. Recent scholarship has identified spatio-temporal patterns in fatal and non-fatal drug overdoses, illuminating the evolving nature of geographic clusters, or “hotspots,” of overdose over time [2,20,23,29]. Critically, much of this research has described spatio-temporal variations in overdose over protracted time intervals (e.g., months, years) or administrative geographies (e.g., census tracts, postal code areas), potentially obscuring micro-temporal (e.g., hours, days) and geosocial (e.g., venue, activity space) heterogeneities in overdoses. Unpacking place-based characteristics of—as well as hour-by-hour, day-to-day, and seasonal variations in—opioid-involved overdoses may facilitate agile allocation and rapid deployment of life-saving medical aid to locations where overdoses cluster over time.

Accordingly, the present study leveraged administrative data to characterize high-dimensional time-space attributes of EMS responses to non-fatal opioid overdoses, as well as layperson naloxone administration during these events, among adults in Rhode Island, which ranks among the highest U.S. states in per-capita overdose deaths [27].

## Methods

Data were derived from timestamped EMS responses to non-fatal opioid-involved overdoses across Rhode Island municipalities between January 1st, 2020, and December 31st, 2022, reported electronically to and compiled by the Rhode Island Department of Health via the Rhode Island Emergency Medical Services Information System (RI-EMSIS) ImageTrend. EMS responses meeting the following validated case definition for opioid-involved overdose events were included: (1) primary or secondary EMS event impression was recorded as overdose-related, and naloxone was administered; (2) primary or secondary EMS event impression was recorded as overdose-related, and naloxone and unresponsive terms were abstracted from the EMS event narrative; (3) naloxone was administered, and medication response was noted as improved; (4) naloxone and at least two different overdose-related terms from five categories (i.e., altered mental status, pupil constriction, decreased respirations, ingestion, overdose) were abstracted from the EMS event narrative; or (5) naloxone was administered before EMS arrival, and the naloxone administration source was documented [15]. Interfacility EMS transfers, overdose events resulting in deaths upon EMS arrival, or EMS runs involving persons 17 years or younger were excluded.

The primary outcome was EMS deployment location, collapsed into the following categories: residential housing; outdoor public spaces (e. g., streets/roads, sidewalks, alleyways, natural areas); businesses (e.g., bars/nightclubs, service stations, restaurants, retail establishments); supervised facilities (e.g., medical facilities, shelters, halfway houses, group homes, jails/prisons); vehicles or parking lots; and hotels/motels. A secondary outcome was naloxone administration source, corresponding to the person who initially administered naloxone during the overdose event (*layperson/self* versus *first responder [EMS or police]*).

Other measured individual and event-level characteristics included age (measured in discrete years); sex (*male* versus *female*); calendar year; season (*spring* versus *summer* versus *autumn* versus *winter*); day of the week, measured in discrete days (Monday-Sunday) and separately dichotomized into weekdays (Monday-Friday) and weekends (Saturday-Sunday); and time of day, collapsed into the following intervals over a 24-h period: early morning (5:00AM-8:59AM), late morning (9:00AM-12:59PM), early afternoon (1:00PM-4:59PM), early evening (5:00PM-8:59PM), late evening (9:00PM-12:59AM), and overnight (1:00AM-4:59AM).

Data were managed and analyzed in Stata/IC 15.1 (StataCorp LLC, College Station, TX). After calculating descriptive sample statistics, we estimated hour-by-hour variations in locations of EMS responses to non-fatal overdoses over the study period, reported as frequencies and proportions. Next, we used multivariable Poisson regression with robust standard errors to identify statistically significant (*p* < 0.05) time-space correlates of EMS responses to overdoses in residential housing and outdoor public spaces, respectively, compared to all other location types. We implemented these same modeling procedures to identify temporal and place-based correlates of layperson naloxone administration, relative to naloxone administration by any first responder. To neutralize potential effect modification by early COVID-19 restrictions on EMS callouts for opioid overdoses, multivariable models were rerun excluding EMS runs between March 16th and June 1st, 2020—aligning with the timing of initial implementation and loosening, respectively, of shelter-in-place ordinances [25,37]. Regression coefficients were reported as adjusted prevalence ratios (adjPR) with 95 % confidence intervals (95 % CI).

## Results

From January 2020 to December 2022, EMS responded to 5377 non-fatal opioid-involved overdoses. Most responses for suspected opioid overdoses were in persons under 55 years (80.6 %, median age: 39 years, interquartile range: 30–51 years) and males (68.7 %). Responses were evenly distributed by year, season, and day of the week; however, EMS runs were most likely to occur between the early afternoon and late evening hours (66.0 %), peaking during the early evening hours (25.4 %). Most EMS responses were to residential housing (61.1 %) and outdoor public spaces (19.3 %), followed by businesses (8.4 %), supervised facilities (5.3 %), vehicles or parking lots (4.3 %), and hotels/motels (1.6 %). In most cases, EMS was the source of naloxone administration (79.0 %), but laypersons (10.6 %) and police (10.4 %) were equally as likely to have administered naloxone.

**Fig. 1.**
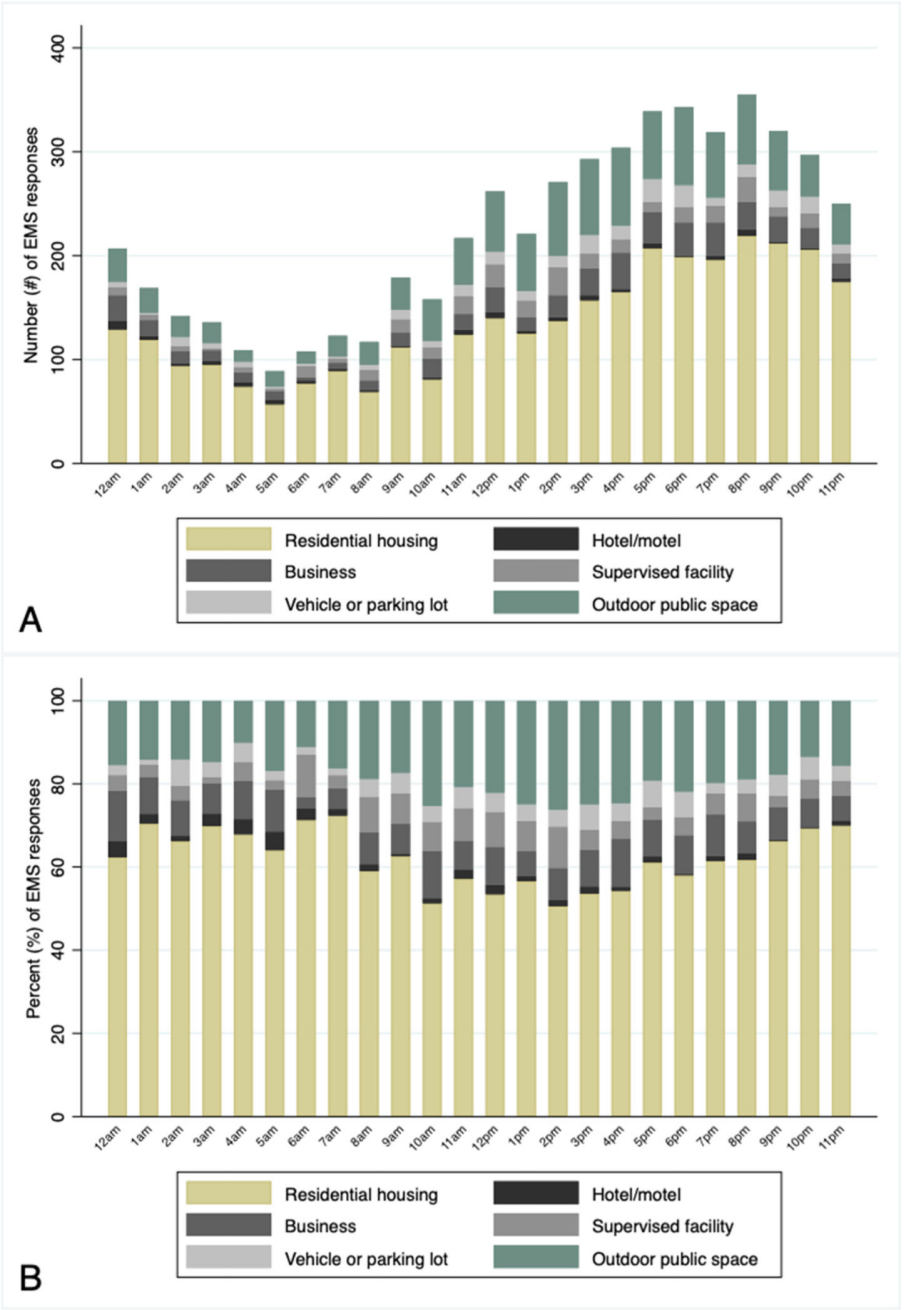
Hour-by-hour frequency (Panel A) and proportion (Panel B) of emergency medical service (EMS) responses to suspected opioid overdoses, by location of suspected overdose.

Fig. 1 visualizes cumulative hour-by-hour frequencies and proportions, respectively, of EMS responses to opioid-involved overdoses between 2020 and 2022. Across panels, EMS runs clustered in residential housing in the late evening, overnight, and early morning hours; however, the fraction of EMS runs for suspected overdoses in outdoor public spaces blipped during daylight hours (late morning through late afternoon).

Table 1 presents time-space correlates of EMS responses to overdoses in residential housing and outdoor public spaces, respectively—relative to all other locations. EMS responses to overdoses in residential housing clustered significantly in the spring (adjPR=1.10, 95 % CI: 1.03–1.17, *p* = 0.003) and winter (adjPR=1.15, 95 % CI: 1.08–1.22, *p* < 0.001) months and were significantly higher in the early morning (adjPR=1.25, 95 % CI: 1.14–1.36, *p* < 0.001), early evening (adjPR=1.14, 95 % CI; 1.06–1.22, *p* < 0.001), late evening (adjPR=1.27, 95 % CI: 1.19–1.36, *p* < 0.001), and overnight (adjPR=1.29, 95 % CI: 1.19–1.40, *p* < 0.001) hours. By comparison, EMS responses to overdoses in outdoor public spaces were significantly elevated in the spring (adjPR=1.22, 95 % CI: 1.03–1.46, *p* = 0.025), summer (adjPR=1.53, 95 % CI: 1.29–1.80, *p* < 0.001), and autumn (adjPR=1.48, 95 % CI: 1.25–1.76, *p* < 0.001) months, as well as the late morning (adjPR=1.65, 95 % CI: 1.30–2.11, *p* < 0.001), early afternoon (adjPR=1.91, 95 % CI: 1.51–2.40, *p* < 0.001), and early evening (adjPR=1.47, 95 % CI: 1.17–1.86, *p* = 0.001) hours. Sensitivity analyses excluding observations between March and June 2020 yielded comparable results.

**Fig. 2.**
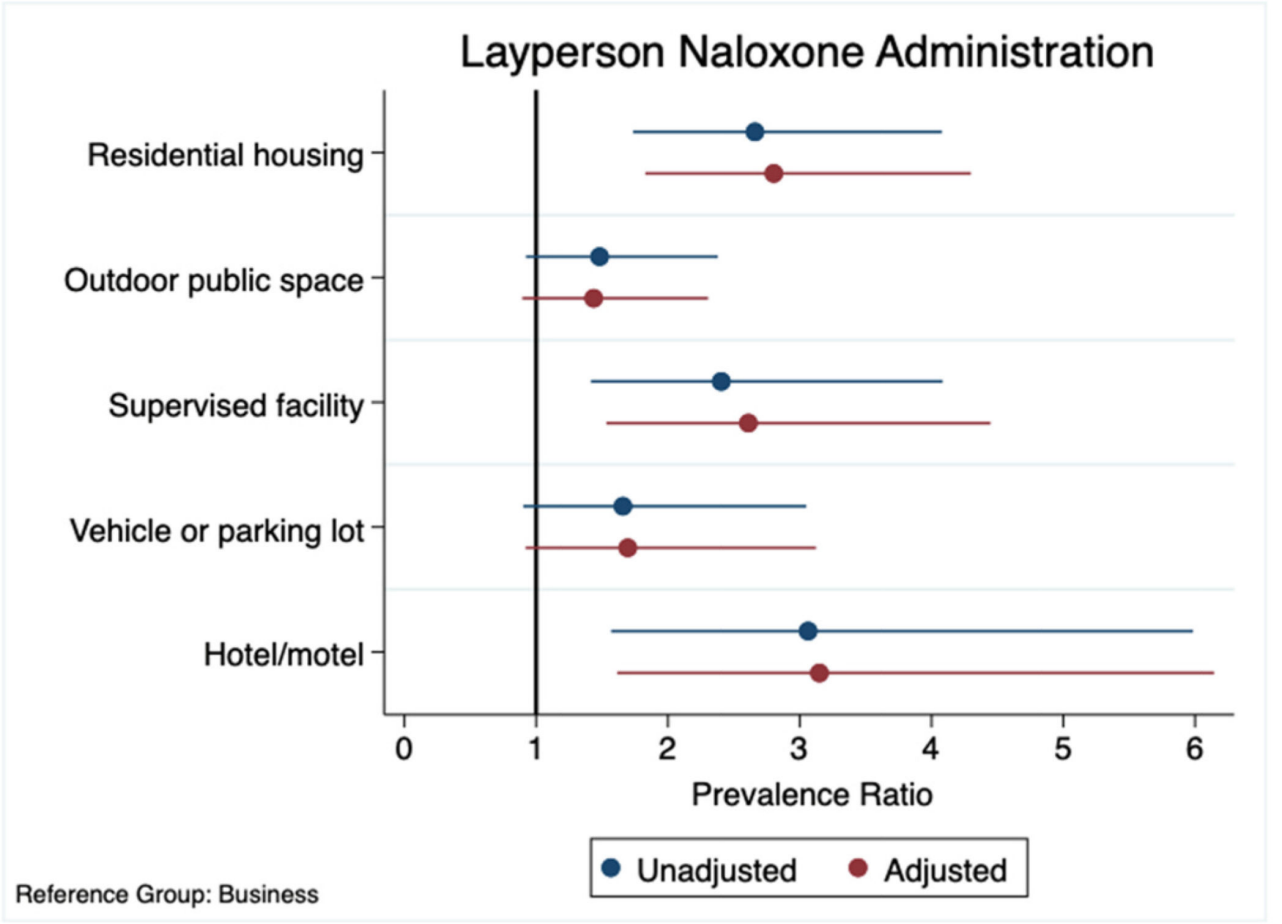
Unadjusted and adjusted prevalence ratios (PR) and 95 % confidence intervals (95 % CI) of naloxone administration by laypersons during emergency medical service (EMS) responses to opioid-involved overdoses, by location of suspected overdose. *Notes*: Prevalence ratios (dots) and 95 % confidence intervals (bars) were obtained from Poisson regression with robust standard errors. Naloxone administration by EMS or police served as the reference group in univariate and multivariable models. The multivariable model was adjusted for age group, sex, year, season, day of the week, and time of day.

Fig. 2 displays place-based correlates of naloxone administration by laypersons during emergency medical service (EMS) responses to opioid-involved overdoses, by location of suspected overdose. Compared to businesses, where the frequency of naloxone administrations by laypersons was lowest (4.7 %), layperson naloxone administration was significantly higher in residential housing (adjPR=2.80, 95 % CI: 1.83–4.30, *p* < 0.001), supervised facilities (adjPR=2.61, 95 % CI: 1.53–4.45, *p* < 0.001), and hotels/motels (adjPR=3.15, 95 % CI: 1.61–6.14, *p* = 0.001). Separately, layperson naloxone administration clustered significantly in the early afternoon hours (adjPR=1.40, 95 % CI: 1.03–1.92, *p* = 0.031) (results not shown).

## Discussion

Across 5377 EMS-attended non-fatal opioid overdoses between 2020 and 2022 in Rhode Island, most occurred in residential housing and in the afternoon-to-evening hours. Building on prior literature characterizing seasonal patterns in opioid overdoses [1,21,26], administrative data leveraged in the present study uncovered granular (i.e., hour-by-hour) time-and-place variations in EMS responses, revealing spatio-temporal heterogeneities in opioid use and non-fatal overdose in communities. These findings convey a dynamic social geography of opioid use, characterized by mobility from private residences to venues/spaces outside the home—primarily outdoor public spaces—during daylight hours. The uptick in EMS responses in the latter half of the day could further reflect temporal variations in overdose risk, attributed potentially to increased opioid use later in the day in response to reemerging withdrawal symptoms [10,13,36].

Despite increased availability and access to naloxone in community settings [16,28], results from the present study suggest layperson naloxone administration during EMS-attended opioid overdoses in Rhode Island is uncommon. In circumstances when laypersons administered naloxone prior to EMS arrival, they were more likely to occur in indoor, housing environments, where bystanders (e.g., family members, friends/peers) are sometimes present and equipped with naloxone [34]. Likewise, layperson naloxone administration during non-fatal overdoses tended to occur in the early afternoon, when individuals may be less likely to use drugs alone [32,38]. Rapid naloxone intervention by laypersons in housing environments, coupled with increased naloxone coverage in non-residential contexts (e.g., businesses, public spaces, encampments), may help avert some EMS callouts for opioid-involved overdoses. Nevertheless, increased naloxone possession alone may be insufficient to increase naloxone intervention by bystanders during overdose events, as pervasive stigma associated with naloxone carrying and discomfort administering naloxone can demotivate naloxone administration by bystanders even in the presence of naloxone availability [3]. Structural interventions mitigating stigma associated with naloxone carrying and bolstering bystander self-efficacy/confidence administering naloxone are imperative to closing this naloxone “possession-administration” gap.

This study is subject to several limitations. First, case definitions for opioid-involved overdoses and other event-related indicators (i.e., naloxone administration by laypersons) were abstracted from unstructured EMS response narratives. Application of this case definition, thus, likely underestimated the true number of EMS responses to opioid-involved overdoses, and event indicators are susceptible to misclassification and missingness. Second, access to administrative data was restricted to a specific set of demographic and event-level attributes, including aggregation of EMS response locations (i.e., private residences) limiting granularity in these data. Other encounter-related characteristics (e.g., EMS response time) and outcomes (e.g., transport to emergency department) reported in other studies could not be assessed here [9,26]. Third, the data leveraged in the present study were derived from administrative records linked to EMS responses. Results, therefore, may have limited transportability to non-fatal opioid-involved overdoses unattended or unwitnessed by EMS, including overdoses where laypersons administered naloxone and successfully revived the individual without ever summoning EMS, which may be motivated by fears of bystander criminalization even in the presence of Good Samaritan laws [22]. Fourth, EMS encounters with the same individual, which are increasingly common [11], could not be identified from the underlying administrative data due to data privacy concerns. Lastly, administrative data only represent EMS encounters for non-fatal opioid overdoses and do not capture fatal opioid overdoses—limiting the generalizability of findings to only EMS-attended, non-fatal overdoses. Future studies could similarly characterize time-and-space variations in fatal opioid overdoses and naloxone intervention by EMS or laypersons.

## Conclusions

Most EMS responses to non-fatal opioid overdoses in Rhode Island from 2020 to 2022 occurred in housing outside of traditional work hours. Our data indicate that spatio-temporal patterns of opioid use require agility in EMS responses to overdose throughout the day. Continued adulteration of the illicit opioid supply by sedative agents like benzodiazepines and, more recently, xylazine reinforce the urgency of earlier detection and response to overdoses, especially by laypersons [14,33]. This could be facilitated with increased naloxone possession in places outside households where overdoses occur (e.g., hotels/motels, businesses), in addition to expanded coverage of overdose detection technologies (e.g., reverse-motion detectors in restrooms) or virtual overdose monitoring services (e.g., digital applications) for people using drugs alone [24,30].

## Figures and Tables

**Table 1:** Unadjusted and adjusted prevalence ratios (PR) and 95 % confidence intervals (95 % CI) of emergency medical service (EMS) responses to residential housing and outdoor public spaces (referent group: all other locations), respectively, for suspected opioid overdoses.

Variables	*EMS response to residential housing*				*EMS response to outdoor public spaces*			
	PR (95 % CI)	*p*-value	adjPR (95 % CI)	*p*-value	PR (95 % CI)	*p*-value	adjPR (95 % CI)	*p*-value
***Individual information*** Age group 18–34 years	1.00	*Ref*.	1.00	*Ref*.	**1.65 (1.38–1.97)**	**< 0.001**	**1.78 (1.49–2.12)**	**< 0.001**
35–54 years	1.04 (0.99–1.09)	0.133	**1.06 (1.01–1.11)**	**0.028**	**1.59 (1.33–1.90)**	**< 0.001**	**1.65 (1.38–1.96)**	**< 0.001**
55 + years Sex	**1.17 (1.10–1.23)**	**< 0.001**	**1.21 (1.14–1.28)**	**< 0.001**	1.00	*Ref*.	1.00	*Ref*.
Male	1.00	*Ref*.	1.00	*Ref*.	**1.43 (1.25–1.63)**	**< 0.001**	**1.41 (1.24–1.60)**	**< 0.001**
Female ***Event characteristics*** Year	**1.14 (1.10–1.20)**	**< 0.001**	**1.14 (1.09–1.19)**	**< 0.001**	1.00	*Ref*.	1.00	*Ref*.
2020	1.03 (0.97–1.08)	0.360	1.03 (0.97–1.08)	0.315	1.00	*Ref*.	1.00	*Ref*.
2021	1.01 (0.96–1.07)	0.666	1.01 (0.96–1.06)	0.666	1.08 (0.94–1.24)	0.263	1.09 (0.95–1.25)	0.196
2022	1.00	*Ref*.	1.00	*Ref*.	1.11 (0.96–1.27)	0.152	1.13 (0.98–1.29)	0.092
Season Spring (March-May)	**1.10 (1.04–1.17)**	**0.002**	**1.10 (1.03–1.17)**	**0.003**	**1.22 (1.02–1.45)**	**0.031**	**1.22 (1.03–1.46)**	**0.025**
Summer (June-August)	1.03 (0.97–1.10)	0.307	1.03 (0.97–1.10)	0.331	**1.53 (1.29–1.80)**	**< 0.001**	**1.53 (1.29–1.80)**	**< 0.001**
Autumn (September-November)	1.00	*Ref*.	1.00	*Ref*.	**1.49 (1.25–1.77)**	**< 0.001**	**1.48 (1.25–1.76)**	**< 0.001**
Winter (December-February)	**1.15 (1.08–1.23)**	**< 0.001**	**1.15 (1.08–1.22)**	**< 0.001**	1.00	*Ref*.	1.00	*Ref*.
Day of the week Weekday (Monday-Friday)	1.00	*Ref*.	1.00	*Ref*.	1.04 (0.92–1.17)	0.533	1.02 (0.91–1.15)	0.702
Weekend (Saturday-Sunday) Time of day	1.03 (0.98–1.08)	0.258	1.02 (0.98–1.07)	0.359	1.00	*Ref*.	1.00	*Ref*.
Early morning (5:00AM–8:59AM)	**1.25 (1.14–1.36)**	**< 0.001**	**1.25 (1.14–1.36)**	**< 0.001**	1.17 (0.87–1.58)	0.307	1.19 (0.88–1.60)	0.256
Late morning (9:00AM–12:59PM)	1.04 (0.96–1.13)	0.301	1.03 (0.95–1.12)	0.431	**1.58 (1.23–2.03)**	**< 0.001**	**1.65 (1.30–2.11)**	**< 0.001**
Early afternoon (1:00PM–4:59PM)	1.00	*Ref*.	1.00	*Ref*.	**1.87 (1.48–2.36)**	**< 0.001**	**1.91 (1.51–2.40)**	**< 0.001**
Early evening (5:00PM–8:59PM)	**1.13 (1.05–1.21)**	**0.001**	**1.14 (1.06–1.22)**	**< 0.001**	**1.48 (1.17–1.87)**	**0.001**	**1.47 (1.17–1.86)**	**0.001**
Late evening (9:00PM–12:59AM)	**1.25 (1.17–1.34)**	**< 0.001**	**1.27 (1.19–1.36)**	**< 0.001**	1.16 (0.90–1.50)	0.250	1.14 (0.89–1.47)	0.294
Overnight (1:00AM–4:59AM)	**1.28 (1.18–1.39)**	**< 0.001**	**1.29 (1.19–1.40)**	**< 0.001**	1.00	*Ref*.	1.00	*Ref*.

Notes: Bolded values represent statistically significant (p < 0.05) prevalence ratios modeled using Poisson regression with robust standard errors. All other locations of suspected overdoses served as the reference groups in univariate and multivariable models for EMS callouts to residential housing and outdoor public spaces, respectively. Multivariable models were adjusted for all variables presented in the table.
